# Multicultural teams: Does national diversity associate with performance in professional soccer?

**DOI:** 10.1371/journal.pone.0325025

**Published:** 2025-06-02

**Authors:** Thadeu Gasparetto

**Affiliations:** West Virginia University, College of Applied Human Sciences. Morgantown, United States of America; Instituto Politecnico de Santarem Escola Superior de Desporto de Rio Maior, PORTUGAL

## Abstract

This study examines whether the national diversity of players in multicultural teams affects the performance of professional soccer clubs. I analyze data from the top-tier leagues of six European countries – England, Belgium, Germany, Cyprus, Latvia, and the Netherlands – covering the 2015/2016–2020/2021 seasons. These leagues were selected because they impose no limits on the number of foreign players in senior squads, providing a clear view of this phenomenon. Using Ordinary Least Squares (OLS) estimations in a forward stepwise approach, I find that national diversity does not correlate with club performance. However, the total number of foreign players is positively associated with performance, with each additional foreign player correlating with a 0.42% increase in win percentage. Moreover, the most complete model also indicates that an additional domestic league title is associated with a 0.99% increase in win percentage per title, while participation in the UEFA Champions League is associated with a 13.6% higher win percentage. Future research should explore the causal mechanisms underlying this relationship, examining how specific attributes of foreign players, such as playing style and experience, contribute to performance. Additionally, studies could investigate how management practices, particularly the adaptation of foreign players to a new country and culture, influence their impact on club success.

## Introduction

The sport’s immense appeal has transformed it into a significant industry where strategic management and investment are crucial [1]. Soccer clubs operate in a highly competitive market, generating revenue through diverse channels such as ticket sales, broadcasting rights, and player transfers [2]. The Bosman ruling of 1995 revolutionized soccer transfers by allowing players to move freely between clubs after their contracts expired, thereby increasing player mobility and altering team dynamics [3–5]. This shift has led to a notable increase in the national diversity of teams, as clubs can now recruit from a global talent pool [6–7].

Research in economics provides insights into how diversity affects organizational outcomes, highlighting the tension between diversity benefits and challenges [8]. While diverse workforces bring different perspectives, they can introduce inefficiencies due to coordination difficulties and communication barriers [9]. In organizational settings, diversity can either enhance creativity and problem-solving or undermine cohesion and productivity, depending on context and management [10].

Empirical research on national diversity’s impact on firm performance shows mixed results. Studies indicate that leaders with multicultural experiences are more effective in managing diverse teams [11], and that experiential diversity’s effects on performance vary, emphasizing the need to balance diversity with integration [12]. These findings are particularly relevant to professional sports, where teams function as high-performance organizations that must integrate diverse individual skills and backgrounds.

In professional soccer, research has found negative effects of cultural diversity on team success in European leagues, suggesting management challenges might outweigh benefits [13]. The relationship between national diversity and performance appears complex and nonlinear, with performance potentially benefiting from diversity beyond certain thresholds [14–15]. While squad diversity could enhance team efficiency, it might simultaneously introduce inefficiencies due to communication costs [16].

A study examining team composition has reported negative effects of fractionalization, defined as the diversity level within a group of foreign players, on team performance [17]. This finding suggests that excessive heterogeneity among foreign players may create additional integration challenges beyond those posed by the mere presence of foreign players.

The influence of national diversity on team performance gained significant academic interest following the Bosman ruling. This phenomenon relates to broader research on high-skilled labor migration, which has demonstrated positive contributions to economic outcomes in wealthier countries [18–20]. Early research in soccer diversity differentiated between effects in defensive and offensive roles, suggesting defensive diversity improved performance through varied strategic insights, while offensive diversity might lead to less coherent play [21].

Cultural and linguistic differences among players can impose substantial transaction costs, potentially lowering team cohesion and performance [22–23]. Analysis of the German Bundesliga revealed weak correlations between demographic diversity and performance, highlighting the need for approaches accounting for contextual influences [14]. Similarly, research on international career diversity in national teams during the FIFA World Cup 2006 illustrated diversity’s dual nature – offering benefits while introducing complexities [12].

Further studies of European soccer leagues demonstrated how high diversity levels could detract from performance in coordination-intensive tasks [13]. The relationship between a team’s predominant nationality and performance in the English Premier League and Spanish La Liga showed nonlinear patterns, with performance initially declining with increasing numbers from the predominant nationality but improving beyond certain thresholds [15].

Research on the Italian Serie A teams revealed negative effects of high fractionalization on performance, attributed to task complexity and team wealth [17]. These findings align with broader organizational research suggesting that diversity can impose significant costs in environments requiring high cohesion and coordination [23–24].

Despite these valuable insights, a significant research gap exists in understanding how national diversity impacts performance, specifically in leagues with minimal restrictions on foreign players. Previous research faced methodological challenges in accurately identifying this effect, as restrictions on foreign players naturally influenced the demand for international talent, creating an artificial ceiling on diversity levels that could be observed and analyzed.

This study aims to address this gap by examining the relationship between national diversity and team performance in leagues with few or no foreign player restrictions. By analyzing data from the Belgian Jupiler Pro League, Cypriot Protathlima Cyta, Dutch Eredivisie, English Premier League, German Bundesliga, and Latvian Optibet Virsliga over six seasons, it investigates whether diverse national composition correlates with improved performance outcomes. The research question specifically examines: To what extent does national diversity impact team performance when regulatory constraints on foreign player recruitment are minimal?

The primary contribution of this paper lies in its focus on leagues with minimal to no restrictions on foreign players, providing a clearer understanding of how unrestricted national diversity affects team performance. This approach allows more accurate assessment of high diversity levels, potentially uncovering dynamics that previous research constrained by foreign player restrictions could not reveal. The findings contribute not only to soccer economics but also offer broader implications for labor market economics by exploring how diversity functions in high-performance, team-based environments. The analysis informs discussions around trade-offs between diversity and cohesion, management of multicultural workforces, and how regulatory frameworks affect foreign talent assimilation in high-skill industries, relevant to economic literature on globalization and labor mobility.

## Methods

### Leagues and foreign players policy

This study explores the impact of national player diversity on soccer club performance by examining leagues with minimal restrictions on foreign players. The selected leagues, each notable for their openness to international talent, are analyzed to assess how varying levels of foreign player participation influence team success.

The Belgian Soccer League (Jupiler Pro League) comprises 18 teams and imposes no restrictions on foreign players, fostering a diverse player base. The Cyprus Soccer League (Protathlima Cyta) has a unique policy requiring teams to declare only two players with Cypriot passports, leading to a high proportion of foreign players across its 12–14 teams. The Dutch Soccer League (Eredivisie), also with 18 teams, imposes no significant limits on foreign players. In the English Premier League, which features 20 teams, few restrictions existed historically; however, since 2010, visa regulations have introduced indirect constraints based on players’ international experience. While there is no direct limit on foreign players, stringent visa requirements ensure that only those with substantial international credentials or high-performance levels can participate. The German Soccer League (Bundesliga), with 18 teams, similarly has minimal restrictions on foreign players, apart from a requirement to register a certain number of German players. The Latvian Soccer League (Optibet Virsliga), with 8–10 teams, has a history of minimal restrictions on foreign players. Recently, it increased the number of foreign players allowed on the field from five to eight, though there is no limit on the total number of foreigners in each squad.

While acknowledging the complexity of player regulations, this study specifically focuses on leagues with minimal direct restrictions on player hiring based on nationality. The English Premier League’s Homegrown Player Rule, for instance, represents an indirect regulatory mechanism that does not entirely prevent foreign player participation but introduces nuanced constraints on squad composition. Unlike direct nationality-based restrictions, this rule allows for significant international player representation, with some clubs demonstrating the ability to field predominantly foreign starting lineups. This approach enables a comprehensive examination of international talent dynamics across different soccer leagues.

The information regarding restrictions on foreign players and the number of participating teams in each selected league was gathered from the official websites of the respective leagues. This ensures the accuracy and reliability of the data, as these sources provide up-to-date and authoritative details about league regulations and team compositions.

### Data and variables

The dataset for this study spans six soccer seasons, from 2015/2016–2020/2021, and includes data from a total of 155 clubs across the selected leagues. Data were manually sourced from Transfermarkt (https://www.transfermarkt.com), due to its extensive and reliable coverage of European soccer statistics.

A total of 786 observations were collected for this study. In this study, each observation represents a club in a given season – the analysis is performed at club-level data, where each club is observed only in seasons when it competed in the top tier of its respective national league. The data encompasses six major soccer leagues: the Dutch Eredivisie, the English Premier League, the Latvian Optibet Virsliga, the Cypriot Protathlima Cyta, the German Bundesliga, and the Belgian Jupiler Pro League. These soccer leagues were selected due to the limited (to no) restrictions on foreign players. The number of teams varied each season due to league regulations, which resulted in promotion and relegation between divisions based on team performance. Consequently, 564 observations were used for the final analysis. This sample size is sufficient to draw robust conclusions, providing valuable insights into the performance dynamics of the selected clubs and leagues.

A linear regression model is employed to analyze the relationship between player diversity and team performance. The dataset includes clubs from top-division leagues in selected countries, with annual data collected for each team. Central to this analysis is the dependent variable Win Percentage, which quantifies the proportion of matches won by a team. This metric is particularly suitable as it directly reflects team success, offering a clear and quantifiable indicator of performance crucial for assessing the impact of player diversity. By calculating the ratio of points earned to total possible points, Win Percentage effectively captures performance variations across different contexts and league structures. This approach aligns with previous research in sports economics [25,26], which also employed similar performance metrics to examine success, thereby reinforcing its relevance in the field.

To ensure a robust analysis, several explanatory variables are included. These are: the total market value of the team, which reflects the financial worth of the squad; the number of players in the team; the number of foreign players, which helps gauge the level of international diversity within the squad; and the number of different nationalities represented in the team, indicating the breadth of international presence. Additionally, the average age of the squad is considered to account for the experience level of the team. Two dummy variables are utilized: Coach ex-player, which indicates whether the coach has prior experience as a professional soccer player, and Local coach, which shows whether the coach is a national of the country where they are employed. The analysis also includes dummy variables for UEFA Champions League participation and domestic league titles, providing insights into the historical success of the teams. The selected explanatory variables are grounded in the theoretical framework of human capital and team dynamics [14,23]. These variables, including total market value and national diversity, are crucial for assessing how financial resources and international composition affect team performance. This approach aligns with previous findings [17], which highlight the significance of diversity in enhancing strategic advantages, thereby providing a robust foundation for analyzing team success in this study.

Fixed effects for season and league are incorporated to control for variations specific to each soccer season and league, ensuring that the results are not confounded by these temporal and geographical factors. Data preparation involved converting certain categorical variables into numeric formats suitable for analysis in Stata/SE 16 (StataCorp, College Station, TX, USA). This preparation included encoding the season, league, and coach nationality to facilitate efficient processing. A dummy variable, Local Coach, was created to examine whether having a coach from the same country as the club impacts performance.

The analysis was based on a sample of 786 observations collected over six seasons, spanning from 2015/2016–2020/2021. The data encompassed six major soccer leagues: the Dutch Eredivisie, English Premier League, Latvian Optibet Virsliga, Cypriot Protathlima Cyta, German Bundesliga, and Belgian Jupiler Pro League. The number of clubs varies over time due to the promotion and relegation system, which results in different clubs participating in the top tier each season; clubs that are relegated do not compete in the top division and, therefore, are not included in the dataset for those seasons. Therefore, the final dataset includes 564 observations.

### Econometric modelling

To investigate the determinants of soccer team performance, a series of Ordinary Least Squares (OLS) regressions were employed, using a forward stepwise approach. This method incrementally introduces variables to the regression models, beginning with a basic model and gradually incorporating additional factors to capture the complexity of team performance. The dependent variable across all models is the winning percentage, which is calculated as the ratio of points earned by a club to the maximum possible points in a given season. Panel data techniques were not utilized in this analysis due to concerns regarding the potential identification issues posed by club-fixed effects. Many of the variables of interest, may be highly correlated with unobserved club characteristics that remain constant over time, thus leading to biased estimators in a fixed-effects framework. In contrast, the application of an Ordinary Least Squares (OLS) regression is more suitable in this context, as it allows for the estimation of the relationships of interest without the confounding influence of club-specific fixed effects, thereby achieving clearer insights into the association between player diversity on team performance.

Models 1 and 2 focus on the aspects of national dispersion within the squad. Model 1 starts by including the number of foreign players as the sole independent variable, providing a foundational analysis of how international talent is associated with team performance. Model 2 builds on this by adding the number of different nationalities within the team, aiming to explore the correlation between cultural and strategic diversity on performance.

Models 3–6 expand the analysis by incorporating key characteristics of the current squad. Model 3 introduces the average squad age to assess how player experience and maturity might relate to team success. Model 4 adds a quadratic term for average squad age, allowing for the examination of potential non-linear effects, such as whether there is an optimal age range for maximizing performance. Model 5 includes the total number of players in the squad to investigate the possible benefits of squad depth. Finally, Model 6 introduces the total market value of the team as a measure of financial resources, which could influence a club’s ability to attract and retain top talent.

The analysis is further refined in Models 7 and 8, which introduce variables related to the team’s coaching staff. Model 7 incorporates a variable indicating whether the coach is of the same nationality as the club’s home country (local coach), exploring the potential advantage of local knowledge and cultural affinity. Model 8 adds a binary variable to capture whether the coach was a former player, aiming to assess if coaching experience derived from playing can positively correlate with team performance.

Models 9 and 10 introduce historical elements to the analysis. Model 9 includes the number of domestic league titles won by the club, allowing for the examination of whether a club’s past successes in its domestic league affect current season performance. Model 10 adds participation in the UEFA Champions League, considering the association between competing at the highest level of European soccer on domestic league performance.

Finally, Models 11 and 12 incorporate fixed effects to control for external influences. Model 11 introduces season-fixed effects to account for time-specific factors such as changes in league rules or economic conditions, ensuring that the results are not biased by season-specific events. Model 12 adds league fixed effects, controlling for differences across various leagues, such as competitiveness, economic environments, and regulatory frameworks, thereby providing a comprehensive understanding of the determinants of team performance.

This stepwise approach, with each set of models progressively adding new dimensions to the analysis, allows for a thorough investigation of the various factors that are associated with soccer team success. The general equation is exhibited as follows:


$$w_{ijt}=\;\beta_{0}+\theta_{1}D_{ijt}+\varphi_{2}S_{ijt}+\vartheta_{3}C_{ijt}+\omega_{4}H_{ijt}+\epsilon_{ijt}$$

where:

w = winning percentage

β_0_ = the constant term

θ, φ, ϑ, and ω = the vector of the coefficients

D = represents the national diversity variables

S = includes the current squad characteristics

C = covers coach-related variables

H = comprises historical performance variables

i = represents a given soccer club

j = represents a given soccer league

t = represents a given season

∊ = is the error term

## Results

The analysis of the determinants of soccer club performance, as captured by the winning percentage, reveals important insights into how various factors are associated with success. Table 1 displays the summary statistics, while Table 2 presents the empirical results, providing an understanding of these determinants.

**Table 1:** Summary statistics.

Variable	Observations^*^	Mean	Std. Deviation	Min	Max
*Win percentage* (%)	564	0.46	0.16	0.04	0.88
*Total Market Value* (€M)	774	540.39	6935.88	0.025	117000
*N Players*	776	35.49	6.08	14	57
*N Foreigners*	776	17.65	7.01	0	41
*N different Nationalities*	776	12.47	4.75	0	28
*Average Squad age* (years)	776	24.25	1.42	17.6	30.1
*Coach Ex-Player* (dummy)	775	0.89	0.32	0	1
*Local Coach* (dummy)	738	0.61	0.49	0	1
*UEFA Champions League* (dummy)	786	0.06	0.25	0	1
*Domestic League Titles*	786	3.35	6.62	0	35
*Season FE* (categorical)	786	3.5	1.71	1	6
*League FE (*categorical)	786	3	1.87	1	6

*The number of observations varies due to the promotion and relegation of clubs as well as based on the data availability.

**Table 2:** Regression outputs. Dependent Variable = Winning Percentage (Points of club *i* divided by maximum number of points in a league *j* in season *t*).

	(Win %)	(Win %)	(Win %)	(Win %)	(Win %)	(Win %)	(Win %)	(Win %)	(Win %)	(Win %)	(Win %)	(Win %)
VARIABLES	Model 1	Model 2	Model 3	Model 4	Model 5	Model 6	Model 7	Model 8	Model 9	Model 10	Model 11	Model 12
N Foreigners	0.00485***	0.00750***	0.00758***	0.00788***	0.00793***	0.00711***	0.00409*	0.00416*	0.00453**	0.00233	0.00242	0.00418**
	(0.00116)	(0.00206)	(0.00206)	(0.00205)	(0.00225)	(0.00225)	(0.00235)	(0.00235)	(0.00199)	(0.00196)	(0.00196)	(0.00199)
N Different Nationalities		-0.00451	-0.00450	-0.00632**	-0.00633**	-0.00551*	-0.00543*	-0.00548*	-0.00532**	-0.00382	-0.00384	-0.00189
		(0.00290)	(0.00290)	(0.00293)	(0.00294)	(0.00294)	(0.00293)	(0.00292)	(0.00248)	(0.00241)	(0.00241)	(0.00247)
Average Squad Age			-0.00315	0.278***	0.278***	0.276***	-0.0150	-0.0181	0.0196	-0.0372	-0.0566	0.0772
			(0.00486)	(0.0856)	(0.0865)	(0.0859)	(0.170)	(0.170)	(0.144)	(0.140)	(0.141)	(0.139)
Average Squad Age^2^				-0.00600***	-0.00602***	-0.00598***	-4.58e-05	-5.73e-06	-0.000609	0.000624	0.00103	-0.00157
				(0.00183)	(0.00185)	(0.00184)	(0.00352)	(0.00351)	(0.00298)	(0.00289)	(0.00291)	(0.00288)
N Players					-8.30e-05	-0.000542	0.000510	0.000540	-0.00204	-0.000869	-0.000921	-0.00379**
					(0.00164)	(0.00164)	(0.00171)	(0.00171)	(0.00146)	(0.00143)	(0.00145)	(0.00156)
Total Market Value						2.41e-06***	2.38e-06***	2.31e-06***	1.72e-06**	1.25e-06*	1.30e-06*	1.68e-06**
						(8.43e-07)	(8.27e-07)	(8.25e-07)	(7.02e-07)	(6.83e-07)	(6.89e-07)	(6.70e-07)
Local Coach							-0.0415***	-0.0412***	-0.0316**	-0.0309**	-0.0304**	-0.0219*
							(0.0146)	(0.0146)	(0.0124)	(0.0120)	(0.0120)	(0.0124)
Coach Ex-Player								0.0475**	0.00740	0.00458	0.00492	0.0274
								(0.0228)	(0.0196)	(0.0190)	(0.0190)	(0.0190)
Domestic League Titles									0.0110***	0.00936***	0.00937***	0.00988***
									(0.000768)	(0.000786)	(0.000788)	(0.000789)
UEFA Champions League										0.127***	0.127***	0.136***
										(0.0205)	(0.0206)	(0.0209)
Season FE	No	No	No	No	No	No	No	No	No	No	Yes	Yes
League FE	No	No	No	No	No	No	No	No	No	No	No	Yes
Constant	0.364***	0.375***	0.450***	-2.797***	-2.801***	-2.763***	0.854	0.860	0.367	0.994	1.234	-0.458
	(0.0234)	(0.0244)	(0.117)	(0.995)	(0.998)	(0.992)	(2.054)	(2.048)	(1.741)	(1.685)	(1.699)	(1.680)
Observations	564	564	564	564	564	564	537	537	537	537	537	537
R^2^	0.030	0.034	0.035	0.053	0.053	0.067	0.068	0.076	0.334	0.379	0.383	0.427
Adjusted R^2^	0.028	0.031	0.030	0.047	0.045	0.057	0.056	0.062	0.332	0.367	0.365	0.404

Standard errors in parentheses *** p < 0.01, ** p < 0.05, * p < 0.1

The Number of Foreign players consistently exhibits a significant positive correlation with team performance across most models. In the final model (Model 12), the coefficient moderates to 0.00418. Given that the average number of foreign players per team in this sample is 17.65, this implies that each additional foreign player is associated with an approximate increase of 0.004 percentage points in the team’s winning percentage. While this effect is statistically significant, its magnitude remains modest. The moderation observed in the final model suggests that although foreign players contribute positively to performance, their impact is not isolated – it is likely shaped by other factors, such as team cohesion, tactical adaptability, and managerial strategies.

The Number of Different Nationalities shows a statistically negative coefficient in Models 2–4, reaching -0.00633 in Model 4, but becomes insignificant in Model 12. With an average of 12.47 different nationalities per team, this suggests that increased diversity may pose challenges in team integration and communication. The insignificance of this variable in Model 12 indicates that when accounting for factors such as market value and historical success, the effect of national diversity on performance is less pronounced. This result also highlights that the influence of nationalities might be overshadowed by other more significant variables in the model.

The Average Squad Age and its squared term exhibit varying associations across models. The coefficient for average age is insignificant in Models 3 and 4 but becomes significant in Model 4 when considering diminishing returns. In the final model, the squared term’s negative coefficient of -0.00157 indicates diminishing returns to age; beyond a certain point, additional years of average age are associated with reduced performance. Nonetheless, the final coefficients are not statistically significant, suggesting that the squad average age is not an impactful determinant of team performance.

The Number of Players shows a negative impact in Model 12, with a coefficient of -0.00379. Given the average squad size of 35.49 players in the current sample, this result suggests that larger squads might face inefficiencies, potentially due to suboptimal use of resources or ineffective squad management. This highlights the importance of managing squad size efficiently, where a more streamlined roster could be more beneficial for team performance.

The Total Market Value demonstrates a positive and significant association with performance in the final model, with a coefficient of 1.68e-06. Considering the mean total market value of 540.39 million €, each additional unit of market value may contribute a very small but statistically significant increase in winning percentage. This underscores the importance of financial investment in player quality and overall squad depth, with wealthier clubs generally having access to better players and resources.

The Local Coach shows a negative coefficient of -0.0219 in Model 12, significant at the 10% level. With 61% of clubs having a local coach, this result suggests a slight disadvantage associated with local coaching. Although not strongly significant, it implies that local coaches might face challenges compared to their international counterparts, potentially due to differences in experience or exposure to diverse tactical approaches.

The Coach Ex-Player presents a non-significant coefficient in Model 8 and does not show a significant association with performance in the final model. This suggests that having a coach with previous playing experience does not significantly affect the winning percentage. With 89% of coaches being former players, this finding challenges the assumption that ex-players necessarily translate their playing experience into successful coaching.

The Domestic League Titles variable demonstrates a strong positive correlation with team performance in the final model, with a coefficient of 0.00988. Given the average number of domestic league titles is 3.35 within the sample, each additional title is associated with a notable increase in the winning percentage. This strong association highlights the importance of historical success in domestic competitions as a key determinant of current performance, reinforcing that a legacy of winning contributes significantly to sustained success.

The UEFA Champions League Participation variable shows a significant positive coefficient of 0.136 in Model 12. With only 6% of clubs participating in this elite competition, the significant positive correlation of participation underscores the prestige and competitive advantage associated with high-profile international tournaments. Clubs that compete in the UEFA Champions League tend to benefit from enhanced visibility and experience, which significantly boosts their performance. Additionally, it is important to note the bi-directional nature of this relationship, as clubs that perform well domestically are more likely to qualify for the UEFA Champions League, thus creating a feedback loop of success and competition.

Season and League Fixed Effects included in Model 12 account for temporal and geographical variations, ensuring that the observed effects of other variables are not confounded by season-specific or league-specific factors. The persistence of significant effects for domestic league titles and UEFA Champions League participation, even with these fixed effects, underscores their central role in explaining team performance. The moderate significance of other variables, such as the number of foreign players and total market value, suggests that while these factors are relevant, they are secondary to the overarching impact of historical success and international competition.

Overall, the results highlight that historical success in domestic leagues and participation in the UEFA Champions League are the most significant variables positively associated with higher soccer club performance. The analysis also reveals that foreign players and total market value are important, but their effects are moderated by other factors. This nuanced understanding provides valuable insights for soccer clubs aiming to enhance their competitive edge and achieve sustained success.

## Discussion

This study explores the effects of team diversity and managerial characteristics on soccer team performance within the context of less regulated environments regarding foreign player limits. This contrasts with many prior studies that have examined more regulated contexts, providing a fresh perspective on the dynamics of team composition and management.

The findings indicate that national diversity within a soccer team does not have a significant association with performance. This result diverges from previous work [13] which found a negative effect of cultural diversity on team performance. They suggested that diverse teams face challenges such as communication barriers and conflicting soccer philosophies. However, this study, which includes teams from leagues with less stringent foreign player regulations, suggests that the impact of national diversity may be less detrimental in a less regulated environment. This may indicate that the negative effects observed in more regulated contexts might be mitigated when teams have more flexibility in managing diverse talent. Another research [15] observed a nonlinear relationship between national diversity and performance, where performance initially declined with increasing diversity but improved beyond a certain threshold. Nonetheless, the current findings do not reveal a significant relationship, suggesting that the threshold effect might not be evident in this sample or that other contextual factors could influence this dynamic.

The impact of managerial experience, particularly related to multicultural backgrounds, was found to be less pronounced in the present study compared to former evidence [11], which reported that broader multicultural experiences enhance leadership effectiveness, especially in managing diverse teams. Their research suggested that managers with extensive multicultural experiences are particularly effective with more multinational teams. The findings, showing a marginal decrease in performance with local coaches, imply that while multicultural managerial experience might still be beneficial, its association is less clear-cut in this sample.

Previous results [14] highlighted the inconsistent and often weak correlations between demographic diversity and team performance, attributing this to confounding factors such as mean demographic attributes and contextual variables. The findings of the current work, which show no significant association between national diversity and team performance, support the view that demographic diversity effects can be context-dependent and may vary based on the specific characteristics of the teams and leagues under study.

The present results also align with some other pieces of evidence [16], which found that the presence of foreign players has a positive effect on performance, though it can introduce inefficiencies. While this work did not explore quadratic relationships, it does support the notion that foreign players can contribute positively to performance in less regulated environments. Therefore, this contrasts with the previous finding [16] that foreign players can increase inefficiency, suggesting that the benefits of foreign players might outweigh the associated communication costs in a less regulated context.

There is a former evidence of a robust negative effect of diversity fractionalization on team performance in the Italian Serie A, highlighting that this negative impact depends on factors such as the nature of the tasks, team wealth, and the experience level of workers [17]. Those findings suggest that while diversity can introduce challenges, these can be exacerbated or mitigated by other team characteristics. The present research, which does not find a significant correlation between national diversity and performance, suggests that in less regulated environments, the negative effects of fractionalization observed by [17] might be less relevant. This could be due to the greater flexibility teams have in managing and integrating diverse players, potentially leading to more effective team dynamics despite higher diversity.

Another work highlights the positive impact of ancestral diversity on national soccer teams’ performance, suggesting that teams with more genetically diverse players perform better [23]. In that context, despite players sharing the same nationality, diversity in their ancestral backgrounds contributes to success. Those findings contrast with the present results, where national diversity within club teams did not show a significant association with performance. However, the positive effect of ancestral diversity in national teams could be extrapolated to club soccer, where the present work found that a higher number of foreign players – representing diverse backgrounds – also positively impacts performance. This suggests that diversity, whether ancestral in national teams or national in club teams, can enhance soccer team performance when managed effectively.

The findings of the present study have significant implications for both soccer management and future research, particularly in the context of the globalized nature of professional soccer. One of the key positive outcomes of this analysis is the observation that a higher number of foreign players within a team correlates positively with team performance. This result suggests that in less regulated environments, where there are fewer restrictions on the recruitment of foreign talent, teams can effectively harness the benefits of a more globally diverse player pool. Taking into account that former research evidenced an improvement in the competitive balance levels within European domestic leagues after the Bosman Ruling [27], easing the current restrictions on foreign players could potentially be beneficial to European soccer.

On the other hand, the potential competitive implications of easing foreign player restrictions present a complex challenge for soccer leagues. Unrestricted recruitment could inadvertently advantage financially powerful clubs, potentially widening the gap between elite and mid-table teams. While this research highlights the benefits of player diversity, the complex dynamics of competitive balance could not be fully addressed within the scope of the current study. This limitation underscores the need for future research to explore the competitive consequences of player recruitment policies more comprehensively.

Additionally, it is important to emphasize that rules such as homegrown player restrictions are typically implemented to support domestic talent development and protect national team performance. These regulations are founded on the assumption that limiting foreign players creates more opportunities for local talent to grow and compete at the highest professional levels. However, recent research challenges the effectiveness of such policies. A study examining the English Premier League revealed that homegrown regulations may produce unintended consequences, with soccer clubs experiencing decreased efficiency after implementation [26]. This controversial result reinforces the need for further empirical research to provide a comprehensive understanding of the potential impacts of nationality-based restrictions across the global soccer industry.

The fact that the number of nationalities within a team did not have a significant impact on performance, while the number of foreign players did, points to an important distinction. It suggests that what matters more is not the variety of national backgrounds per se, but rather the ability to integrate and leverage the unique skills and experiences that foreign players bring to the team. This insight underscores the importance of focusing on the quality and fit of foreign players, rather than merely aiming for diversity in nationalities. Furthermore, the budgetary constraints and financial capacity of clubs may play a crucial role in player recruitment. In leagues with lower financial resources, clubs with more successful track records may have greater funds to recruit foreign talent, while less successful clubs might mostly rely on domestic players. This dynamic could introduce a reverse association, where a club’s financial capacity influences both the number of foreign players and its performance. Recent research has also demonstrated that wage dispersion can impact clubs differently depending on their relative payroll [28], suggesting that the financial landscape of a league or an individual club might similarly affect recruitment strategies and, consequently, team performance.

While the findings highlight the potential benefits of foreign player diversity, it is important to recognize that successful international player integration involves more than simply increasing the number of foreign players. Factors such as psychosocial adaptation, team cohesion, tactical compatibility, and individual player integration can significantly influence team performance. These critical aspects of player assimilation represent important considerations that the current dataset and methodology cannot fully explore. Further research investigating how clubs facilitate cultural integration, develop communication strategies, and address the challenges of international player recruitment is encouraged. Understanding these mechanisms could provide valuable insights into the underlying factors that contribute to effective team composition and performance.

Moreover, the present findings highlight the critical role of sporting attributes in driving team performance. Variables such as market value, the number of domestic league titles, and participation in European tournaments were found to be significant contributors to higher performance levels. These factors are indicative of a team’s overall strength and experience, suggesting that success is closely tied to both the quality of the players and the historical achievements of the club. High market value often reflects a team’s investment in top talent, while success in domestic and European competitions demonstrates the ability to compete at the highest levels. Together, these attributes reinforce the importance of building a team with proven expertise and experience.

Given these insights, there are clear managerial implications for soccer clubs and leagues worldwide. For leagues that currently impose strict limits on the number of foreign players, the results suggest that easing these restrictions could be beneficial. By allowing clubs to recruit more foreign players, leagues could attract a higher caliber of talent, which in turn could enhance the overall quality of the league. This could lead to increased competition, greater fan interest, and ultimately, higher revenues. As professional soccer continues to become more globalized, leagues that embrace this diversity may find themselves better positioned to succeed in the increasingly competitive international landscape. Nonetheless, the challenges discussed above, such as the potential reduction of playing time for domestic players, the dominance of a small group of clubs, and the complexities regarding the correct integration of foreigners into a new country, indicate that this research topic is not closed and further research should deeper explore these aspects.

For future research, these findings open up several avenues for exploration. It would be valuable to investigate how different leagues with varying levels of foreign player restrictions compare in terms of performance, fan engagement, and financial success. Additionally, further research could explore the mechanisms through which foreign players contribute to team success – whether through technical skills, leadership, strategic insights, or other factors. Understanding these dynamics could help clubs and leagues optimize their recruitment strategies, ensuring that they not only attract top talent but also integrate it in ways that maximize team performance.

While this study provides valuable insights into the effects of foreign player presence and national diversity on team performance within European soccer, the generalizability of the findings to other regions and contexts remains a limitation. The focus on top-tier European leagues, where foreign player restrictions are often more relaxed, may not fully capture the dynamics in more regulated environments, such as leagues with stricter foreign player limits or different socio-economic and cultural settings. For instance, in regions like South America, Africa, or Asia, the effects of foreign players may be more nuanced, potentially shaped by different organizational structures, market conditions, and local preferences. Future research should explore these dynamics in diverse leagues, accounting for the regulatory and cultural differences that may influence team performance.

Furthermore, a key limitation of the present study is the inability to establish causal relationships between foreign player integration, national diversity, and team performance. The analysis highlights significant associations, but it remains unclear whether foreign players directly drive performance improvements or if successful teams, with stronger performance records, are more likely to recruit better foreign talent. The possibility of reverse causality or bi-directional effects cannot be ruled out, where clubs with better financial resources and greater success may be more inclined to bring in foreign players, which, in turn, boosts their performance. While this limitation does not undermine the value of the present work, as the significant associations identified point to potential avenues for clubs and researchers to further explore these dynamics, future studies can provide a more rigorous examination of causality in this context.

## Conclusions

This study provides important insights into how foreign players and national diversity influence team performance in professional soccer. The findings suggest that the presence of foreign players has a significant positive impact on team success, while national diversity alone does not appear to have a comparable effect. This underscores the importance of integrating and leveraging the unique skills and experiences that foreign players bring to a team, rather than merely pursuing diversity for its own sake.

While the research contributes to the understanding of foreign player dynamics in less regulated environments, it also highlights important limitations, such as the inability to confirm causal relationships and the generalizability of the results to other soccer cultures. Future research should address these limitations by exploring different league contexts, longer time periods, and match-level data to deepen the understanding of how foreign talent and diversity affect team performance across various settings.

## Supporting information

S1 Data Research dataset. (DTA)

## References

[1] Sheth H, Babiak KM. Beyond the game: perceptions and practices of corporate social responsibility in the professional sport industry. J Bus Ethics. 2009;91(3):433–50. doi: 10.1007/s10551-009-0094-0

[2] Szymanski S, Smith R. The english football industry: profit, performance and industrial structure. Int Rev Appl Econ. 1997;11(1):135–53. doi: 10.1080/02692179700000008

[3] Antonioni P, Cubbin J. Eur J Law Econ. 2000;9(2):157–73. doi: 10.1023/a:1018778718514

[4] Ericson T. The bosman case. J Sports Econ. 2000;1(3):203–18. doi: 10.1177/152700250000100301

[5] Feess E, Muehlheusser G. Transfer fee regulations in European football. Eur Econ Rev. 2003;47(4):645–68. doi: 10.1016/s0014-2921(02)00308-2

[6] Goddard J, Sloane PJ, Wilson JOS. The bosman ruling and labor mobility in football (Soccer). In: Kahane LH, Shmanske S, editors. The oxford handbook of sports economics: the economicsof sports. Vol. 1. Oxford University Press. 2012. p. 260–80. doi: 10.1093/oxfordhb/9780195387773.013.0014

[7] Rao Sahib P. Status, peer influence, and racio-ethnic diversity in times of institutional change: an examination from European labour law. J Bus Ethics. 2013;126(2):205–18. doi: 10.1007/s10551-013-1936-3

[8] Milliken FJ, Martins LL. Searching for common threads: understanding the multiple effects of diversity in organizational groups. Acad Manag Rev. 1996;21(2):402. doi: 10.2307/258667

[9] Alesina A, La Ferrara E. Ethnic diversity and economic performance. J Econ Lit. 2005;43(3):762–800. doi: 10.1257/002205105774431243

[10] Boone C, Lokshin B, Guenter H, Belderbos R. Top management team nationality diversity, corporate entrepreneurship, and innovation in multinational firms. Strateg Manag J. 2018;40(2):277–302. doi: 10.1002/smj.2976

[11] Lu JG, Swaab RI, Galinsky AD. Global leaders for global teams: leaders with multicultural experiences communicate and lead more effectively, especially in multinational teams. Organ Sci. 2022;33(4):1554–73. doi: 10.1287/orsc.2021.1480

[12] Ruigrok W, Greve P, Engeler M. International experiential diversity and performance at project organizations. Sport Bus Manag Int J. 2011;1(3):267–83. doi: 10.1108/20426781111162675

[13] Maderer D, Holtbrügge D, Schuster T. Professional football squads as multicultural teams: cultural diversity, intercultural experience, and team performance. Int J Cross Cult Manag. 2014;14(2):215–38. doi: 10.1177/1470595813510710

[14] Nüesch S. Are demographic diversity effects spurious?. Econ Anal Policy. 2009;39(3):379–88. doi: 10.1016/s0313-5926(09)50034-3

[15] Tovar J. Performance, diversity and national identity evidence from association football. Econ Inq. 2019;58(2):897–916. doi: 10.1111/ecin.12861

[16] Boto‐García D, Varela‐Quintana C, Muñiz A. Foreign players, team production, and technical efficiency: evidence from European soccer. Bull Econ Res. 2023;75(4):1209–41. doi: 10.1111/boer.12407

[17] Addesa F, Pazzona M, Rossi G. Migrant diversity and team performance in a high‐skilled labour market. Kyklos. 2022;75(3):365–84. doi: 10.1111/kykl.12299

[18] Ager P, Brückner M. Cultural diversity and economic growth: evidence from the US during the age of mass migration. Eur Econ Rev. 2013;64:76–97. doi: 10.1016/j.euroecorev.2013.07.011

[19] Alesina A, Harnoss J, Rapoport H. Birthplace diversity and economic prosperity. J Econ Growth. 2016;21(2):101–38. doi: 10.1007/s10887-016-9127-6

[20] Bove V, Elia L. Migration, diversity, and economic growth. World Dev. 2017;89:227–39. doi: 10.1016/j.worlddev.2016.08.012

[21] Ben‐Ner A, Licht J, Park J. Bifurcated Effects of place‐of‐origin diversity on individual and team performance: evidence from ten seasons of German soccer. Ind Relat. 2017;56(4):555–604. doi: 10.1111/irel.12188

[22] Easterly W, Levine R. Africa’s growth tragedy: policies and ethnic divisions. Quart J Econ. 1997;112(4):1203–50. doi: 10.1162/003355300555466

[23] Beine M, Peracchi S, Zanaj S. Ancestral diversity and performance: evidence from football data. J Econ Behav Organ. 2023;213:193–214. doi: 10.1016/j.jebo.2023.07.024

[24] Lazear EP. Globalisation and the market for team‐mates. Econ J. 1999;109(454):15–40. doi: 10.1111/1468-0297.00414

[25] Flores R, Forrest D, Tena JD. Impact on competitive balance from allowing foreign players in a sports league: evidence from European soccer. Kyklos. 2010;63(4):546–57. doi: 10.1111/j.1467-6435.2010.00487.x

[26] Shin SM, Chung K, Kim C. The effects of homegrown rule on efficiency of sports teams: evidence from the English premier league. Serv Sci. 2024;16(3):143–54. doi: 10.1287/serv.2023.0044

[27] Carmichael F, Thomas D, Ward R. Production and efficiency in association football. J Sports Econ. 2001;2(3):228–43. doi: 10.1177/152700250100200303

[28] Gasparetto T, Barajas A. Wage dispersion and team performance: the moderation role of club size. J Sports Econ. 2021;23(5):548–66. doi: 10.1177/15270025211067793

